# Evaluation of the eruption of permanent teeth and their association with malocclusion

**DOI:** 10.1002/cre2.544

**Published:** 2022-02-14

**Authors:** Anita Fekonja

**Affiliations:** ^1^ Department of Orthodontics Healthcare Centre Maribor Maribor Slovenia; ^2^ Faculty of Medicine University of Maribor Maribor Slovenia

**Keywords:** eruption time, malocclusion, pediatric dentistry

## Abstract

**Objectives:**

This study was done to determine the timing of eruption of permanent teeth by sex and the number of permanent teeth erupted at different ages and to assess its association with malocclusion.

**Material and Methods:**

The sample for this study consisted of 633 healthy subjects (317 boys and 316 girls) aged between 5.0 and 15.0 years. The subjects were divided into subgroups considering an age interval of 1 year.

**Results:**

The mean eruption times were lower for girls compared with boys except for the maxillary and mandibular second premolars and second molars, which were found to erupt earlier in boys. All the permanent mandibular teeth, except the second premolars, tended to erupt earlier than their maxillary antagonists in both sexes. The sequence of eruption differs between girls and boys in the maxillary canine and maxillary second premolars. In Class III malocclusion, all the permanent mandibular teeth erupt earlier than their maxillary antagonists. In the entire sample, the correlation between the number of erupted teeth and age was strong (*p* < .05), but the differences between sexes were not significant.

**Conclusions:**

It is important to know the normal eruption time of permanent teeth in the population due to diagnosis and better treatment planning in pediatric dentistry and orthodontics.

## 1 INTRODUCTION

Anthropology is a multidiscipline science that examines human beings as a part of nature in a defined environment and time. Tooth eruption is a continuous biological process by which developing teeth emerge through the jaws and the overlying mucosa to enter into the oral cavity (Avery, 1992). Although this process is genetically determined (Wise et al., 2002), it has been reported that tooth eruption time, as well as the sequence of tooth eruption, varies with races, geographical location, ethnic, sex, and socioeconomic status (Almonaitiene et al., 2010; Bilewitz & McGregor, 1975; Khan et al., 2006; Mugonzibwa et al., 2002; Nizam et al., 2003; Nonaka et al., 1990; Pahkala et al., 1991). Generally, permanent teeth have been found to erupt between the ages of 5–13, except the third molars (Agarwal et al., 2004; Almonaitiene et al., 2010; Bilewitz & McGregor, 1975; Khan et al., 2006; Mugonzibwa et al., 2002; Nizam et al., 2003; Pahkala et al., 1991; Premik et al., 1994).

Adequate knowledge of permanent tooth eruption time and sequence are important factors for diagnosis and treatment planning in pediatric dentistry and orthodontics as well as community medicine for monitoring the growth of children (Mugonzibwa et al., 2002). As a variety of factors relate to tooth emergence, standards for the emergence of permanent teeth are most useful when they derive from the population to which they are applied.

The purpose of this study was to determine the age of eruption of permanent teeth by sex and malocclusion in children aged 5–15 years, and the number of teeth erupted at different ages, in Maribor, Slovenia.

## 2 METHODS

The study was reviewed and approved by the Institutional Review Board. Informed consent approval was obtained from each patient.

The sample for this study consisted of 633 healthy subjects (317 boys and 316 girls) from the Healthcare Centre Maribor, aged 5–15 years. The subjects were divided into subgroups considering an age interval of 1 year. The distribution of the subjects in the study according to age, sex and malocclusion is presented in Table 1.

**Table 1:** The distribution of subjects by age group, sex, and malocclusion

	Sex								
	Boys (*N* = 317)			Girls (*N* = 316)			Total (*N* = 633)		
Malocclusion	Class I	Class II	Class III	Class I	Class II	Class III	Class I	Class II	Class III
Age									
5	3	4	5	4	4	4	7	8	9
6	5	5	4	3	5	4	8	10	8
7	10	12	11	11	11	13	21	23	24
8	10	9	10	10	9	9	20	18	19
9	10	12	10	13	12	15	23	24	25
10	12	13	13	12	10	12	24	23	25
11	12	11	11	11	9	10	23	20	21
12	12	12	12	11	11	10	23	23	22
13	12	11	11	11	12	12	23	23	23
14	11	12	11	12	10	12	23	22	23
15	8	7	6	7	9	8	15	16	14
Total	105	108	104	105	108	110	210	210	213

Subjects with syndromes, lip/palate clefts, local conditions, and systemic diseases that may influence tooth eruption were not included in the study. Third molars were not included in the study because of their great variability and rare presence before age teen. Aasheim's (1993) study included a panoramic radiograph examination to determine the congenitally missing teeth. The previous study discussed the impact of congenitally missing teeth on the mean eruption times and concluded that agenesis may lead to delayed eruption time. Subjects with congenitally missing teeth were also not included in the present study.

All the subjects were examined by a single examiner (AF). The examination was done using a standard mouth mirror and a probe with adequate natural illumination. The examination commenced from the maxillary right quadrant, looking for the presence of permanent teeth, followed by the maxillary left, mandibular left, and mandibular right quadrants. The number of permanent teeth that erupted in the oral cavity of each child was recorded in his/her file. A tooth with any of its parts that emerged through the gingiva was considered to have erupted. A similar procedure was carried out for all the 633 children included in the study. Each permanent tooth was recorded using the two‐digit notation system of the Fedération Dentaire Internationale.

Skeletal malocclusion was determined by the analysis of cephalometric standards for Slovenians (Drevenšek et al., 2005). All radiographs were taken using the same equipment (Planmeca Promax) by an experienced dental radiology engineer under standard conditions (subjects were in standing position and adequately protected, the Frankfort horizontal plane parallel to the floor, and with the teeth in the maximal intercuspation (centric occlusion). The distance from the focus to the median sagittal plane of the subject's head, and from the median sagittal plane of the head to the film was identical for each patient. The exposure parameters 64 kV and 8 mA were selected. All selected LC fulfilled the inclusion criteria of standard images of good quality, without any grade of exposure or positioning errors. Morphological craniofacial characteristics were evaluated from the digital LC by the orthodontist examiner (A. F.) using the Planmeca Romexis cephalometric software program, for each patient twice at different times to eliminate errors and the mean parameters were calculated.

### 2.1 Statistical analysis

The data were analyzed using the Statistical Package for Social Science Inc. (SPSS, version 10.0 for windows). The mean age (mean value ± SD) of eruption of individual permanent teeth according to sex was calculated. For each sex, the percentages of subjects in whom the tooth was present at specific age levels were determined and transformed into percentile curves. Two sample *t‐test* for independent samples was used to test differences between the mean age of eruption between sex, upper and lower jaws, and right and left sides. *χ* ^2^ test was used to analyze the correlation between age of teeth eruption, malocclusion, and sex.

## 3 RESULTS

A total of 633 children aged 5.0–15.0 years were included in the study. There was no statistically significant difference in age between the sexes (*p* = .90) and in malocclusions between the sexes (*p* = .84) (Table 1).

The mean value ± SD of eruption dates (for emergence) of permanent teeth for boys and girls are shown in Table 2. Generally, the mean eruption times for girls were lower compared with boys except for the maxillary and mandibular second premolars and maxillary and mandibular second molars, which were found to erupt earlier in boys (Table 2). Statistically significant differences were related to the maxillary first premolars (*p* = .041) and mandibular canines (*p* = .017), the girls showing more advanced eruption stages.

**Table 2:** Mean ± SD eruption ages (years) of the maxillary and mandibular permanent teeth

	Sex			
	Boys		Girls	
Tooth	Right, mean ± SD	Left, mean ± SD	Right, mean ± SD	Left, mean ± SD
Upper teeth				
Central incisor	6.61 ± 0.51	6.58 ± 0.52	6.42 ± 0.53	6.57 ± 0.53
Lateral incisor	7.89 ± 0.91	7.86 ± 0.67	7.74 ± 0.76	7.85 ± 0.61
Canine	10.85 ± 0.90	10.88 ± 0.94	10.67 ± 1.07	10.68 ± 0.92
First premolar	10.25 ± 1.06	10.23 ± 1.04	9.91 ± 1.33	9.89 ± 1.31
Second premolar	10.73 ± 1.23	10.69 ± 1.10	10.91 ± 1.36	10.94 ± 1.11
First molar	6.27 ± 0.51	6.29 ± 0.49	6.14 ± 0.33	6.19 ± 0.42
Second molar	12.11 ± 1.15	12.25 ± 1.12	12.48 ± 1.11	12.58 ± 1.13
Lower teeth				
Central incisor	6.08 ± 0.48	6.02 ± 0.41	5.98 ± 0.74	5.92 ± 0.74
Lateral incisor	7.19 ± 0.82	7.12 ± 0.63	7.04 ± 0.97	7.06 ± 0.71
Canine	9.59 ± 0.50	9.71 ± 0.47	9.28 ± 0.58	9.29 ± 0.59
First premolar	10.08 ± 0.84	10.01 ± 0.87	9.89 ± 1.25	9.76 ± 1.12
Second premolar	10.68 ± 1.19	10.96 ± 1.07	10.88 ± 1.19	11.02 ± 1.35
First molar	5.89 ± 0.59	5.59 ± 0.61	5.71 ± 0.49	5.63 ± 0.49
Second molar	11.95 ± 1.15	11.86 ± 1.17	12.03 ± 1.37	12.07 ± 1.66

All the permanent mandibular teeth, except the second premolars, tended to erupt earlier than the maxillary teeth in both sexes. The differences were statistically significant (*p* < .05) for all teeth except the first and second premolars and second molars.

In the present study, the sequence of eruption for maxillary canines and second premolars differs between sexes. The maxillary canine can be expected before the second premolar in 71% and 22% of girls and boys, respectively (*p* < .05).

The differences in the mean eruption ages between right and left contralateral teeth were not significant (*p* > .05).

Table 3 shows the mean value ± SD of eruption dates of permanent teeth according to skeletal Class I, Class II or Class III malocclusion. In Class III malocclusion, all the permanent mandibular teeth erupt earlier than the maxillary teeth. The differences were statistically significant (*p* < .05) for all teeth except the second premolar and second molar. The mean eruption times for all the permanent mandibular teeth in Class III malocclusion were lower compared with Class I and Class II malocclusion except for the mandibular second premolars and second molars. The mean eruption times for all the permanent maxillary teeth in Class II malocclusion were lower compared with Class I and Class III malocclusion except for the maxillary canine and first molar.

**Table 3:** The mean value ± SD of eruption dates (for emergence) of permanent teeth according to skeletal Class I, Class II, or Class III malocclusion

	Malocclusion		
Tooth	Class I, mean ± SD	Class II, mean ± SD	Class III, mean ± SD
Upper teeth			
Central incisor	6.65 ± 0.58	6.14 ± 0.69	6.35 ± 0.48
Lateral incisor	8.06 ± 0.80	7.48 ± 0.56	7.57 ± 0.65
Canine	10.62 ± 0.67	10.73 ± 1.18	11.02 ± 1.15
First premolar	10.10 ± 0.98	9.96 ± 1.19	10.21 ± 1.32
Second premolar	10.61 ± 0.56	10.57 ± 1.23	10.83 ± 1.46
First molar	6.02 ± 0.56	6.33 ± 0.51	6.45 ± 0.69
Second molar	12.19 ± 0.87	12.12 ± 1.33	12.25 ± 0.46
Lower teeth			
Central incisor	6.44 ± 0.53	5.95 ± 0.46	5.82 ± 0.47
Lateral incisor	7.24 ± 0.49	6.93 ± 0.63	6.14 ± 0.83
Canine	10.11 ± 0.89	9.88 ± 0.94	9.33 ± 1.30
First premolar	10.17 ± 0.74	10.06 ± 1.09	9.92 ± 1.25
Second premolar	10.02 ± 0.65	10.59 ± 1.25	10.78 ± 1.32
First molar	5.95 ± 0.69	6.29 ± 0.49	5.50 ± 0.42
Second molar	11.64 ± 0.61	12.31 ± 1.12	12.03 ± 0.53

**Figure 1:**
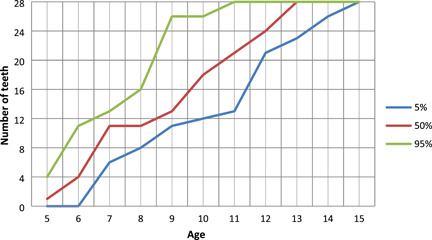
The number of erupted permanent teeth at a certain age in boys

**Figure 2:**
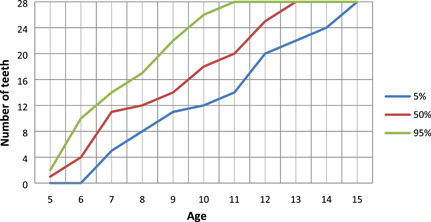
The number of erupted permanent teeth at a certain age in girls

The results of the number of erupted permanent teeth at a certain age are presented by percentile curves (Figures 1 and 2). The curves show the number of erupted teeth for both sexes between the ages of 5.0 and 15.0. For example, at age 8 in 50% of boys and girls, eleven and twelve teeth were erupted, respectively, and at age 12 in 50% of boys and girls, twenty‐four and twenty‐five teeth were erupted, respectively. Children who had the top 5% highest number of erupted teeth are arranged above the upper line (95th percentile) and, children who had the lowest 5% number of erupted teeth are arranged below the lower line (5th percentile). In the entire sample, the correlation between the number of erupted teeth and age was strong (*p* < .05), but the differences between sexes were not significant.

## 4 DISCUSSION

Permanent tooth eruption is genetically determined and influenced by many socioeconomic and environmental factors (Almonaitiene et al., 2010; Wise et al., 2002). The prediction of the timing of dental eruption is useful in interceptive guidance of occlusion, especially to determine eventual extractions of deciduous teeth and timing of orthodontic treatment (Eskeli et al., 2016; Posen, 1965). It is important to know the normal eruption time of teeth in a population.

The current study results reveal some agreement and some disagreement with those of previous studies.

Previous studies by other authors have reported that Caucasians have a delayed time of eruption compared with Africans and people from the Caribbean (Almonaitiene et al., 2010; Bilewitz & McGregor, 1975; Mugonzibwa et al., 2002). Some authors have reported differences in the timing of the permanent teeth eruption in the same population (Eskeli et al., 2016; Leroy et al., 2003; Pahkala et al., 1991).

The study by Eskeli et al. (2016) carried out over the past few decades showed that the permanent teeth of the first phase of mixed dentition erupt earlier at present than 20 years ago. In contrast, the permanent teeth of the second phase of mixed dentition seemed to erupt later, but without statistically significant difference. The timing of permanent teeth eruption in Slovenia was studied more than 30 years ago (Premik et al., 1994). Over that period, many environmental and socioeconomic changes have taken place (Almonaitiene et al., 2010). In the present study, it was found that in boys, most teeth (except upper first and second premolars, and lower second molars) erupt earlier compared with the previous study in the same population, but in girls, the maxillary canines, maxillary first and second premolars, and maxillary second molars, as well as the mandibular second premolars and mandibular second molars, erupted slightly later in the present sample than reported in the previous study in Slovenian children 30 years ago (Premik et al., 1994). One reason for the later emergence of these teeth in the present sample may be the highly improved dental health of Slovenian children. The premature loss of deciduous molar due to caries and, consequently, its accelerative effect on tooth eruption, has decreased. This is in correlation with Eskeli et al. (2016) who also reported that premolars erupted later than previously reported for the Scandinavian population. The biggest difference in eruption times between the present study and the previous one (Premik et al., 1994) in boys was 0.75 years for mandibular canines while among girls, it was 0.85 years for the mandibular second molars.

In the present study, the results showed that the mean eruption times in girls were generally lower, except for the maxillary second premolars and second molars and mandibular second premolars and second molars than in boys. This is in agreement with the study by Moslemi (2004) who reported earlier eruption of permanent teeth in girls, with exception of maxillary second premolars. Kochhar and Richardson (1998) reported earlier emergence of second molars in boys than in girls and explained this phenomenon as a catch‐up development by the age of eruption of the second molars because of the later onset of puberty in the boys. In most teeth, there was a wider range of variation in the eruption dates in girls. The difference in eruption time between boys and girls has been found to vary from one to five months depending on the tooth. The standard deviation related to premolars is higher because it depends on caries and premature loss of the primary molars (Eskeli et al., 2016). These more advanced eruption stages in girls than in boys is in agreement with previous studies in different populations (Dahiya et al., 2013; Diamanti & Townsend, 2003; Eskeli et al., 2016; Kochhar & Richardson, 1998; Leroy et al., 2003; Moslemi, 2004; Mugonzibwa et al., 2002; Nizam et al., 2003; Pahkala et al., 1991). This difference has been attributed to the earlier physical development and maturation of girls. Similarly, in the previous study of the Slovenian population, eruption times were slightly earlier in girls than in boys (Premik et al., 1994).

Permanent teeth usually begin to erupt around the age of six. In the present study, the first permanent teeth to erupt were the first molars in both jaws. They erupt in the maxilla at the age 6.28 ± 0.51 and 6.17 ± 0.37 in boys and girls, respectively, and in the mandible at age of 5.79 ± 0.60 and 5.76 ± 0.49 in boys and girls, respectively. This finding was comparable with the study by Nizam et al. (2003) and Agarwal et al. (2004) who found that the first permanent teeth to erupt were also the maxillary and mandibular first molars but in disagreement with study by Nonaka et al. (1990) and Nyström et al. (2001) who found that the mandibular central incisor erupted earlier than mandibular first molar in both genders.

In the present study, the sequence of eruption differs between girls and boys for maxillary canines and second premolars. In girls, the maxillary canine can be expected before the second premolar, while in boys that order is reversed. This is in agreement with Moslemi (2004) and Leroy et al. (2008) Knowledge about eruption time for the maxillary canine and premolar is critical for dental treatment planning, especially in orthodontic cases of a space deficit in the canine region. According to Knott and Meredith (1966) the fourth tooth to commonly erupt is the first premolar in the maxilla and the canine in the mandible.

There is also a symmetry in the right and left sides reflecting the symmetry of the whole body. We found no statistically significant differences in the mean eruption times between the contralateral teeth on the right and left sides of the jaws. This finding is in agreement with other studies (Bilewitz & McGregor, 1975; Leroy et al., 2003) that reported that differences between the emergence ages of contralateral teeth were not statistically significant. Statistically significant differences in the eruption of contralateral permanent teeth were reported by Rajić et al. (2000).

The results of this study also showed that the mean eruption times of most permanent teeth was earlier in the mandible than the maxilla in both sexes, which is in agreement with some other studies (Diamanti & Townsend, 2003); Kochhar & Richardson, 1998; Leroy et al., 2003; Moslemi, 2004; Mugonzibwa et al., 2002; Nizam et al., 2003).

The distribution of children according to the number of erupted permanent teeth per age (years) is shown in percentile curves. In the present study, the number of erupted teeth in boys and girls were higher in age five, six, and seven than previously recorded in Slovenian children 30 years ago. At the age of eight and later, the number of erupted teeth is comparable with the previous result of Premik et al. (1994).

Ogodescu et al. (2011) reported significant differences of erupted teeth between boys and girls aged 9.5–10.4 years (girls have a mean number of 17.82 teeth compared with 15.23 in boys; *p* = .007) and 11.5–12.4 years (the mean number of 25.45 teeth for girls and 23.00 for boys). In the present study, girls have more teeth erupted at ages 8.0–9.0 and 11.0–12.0, but without statistically significant differences between sexes. The graphs of percentile curves serve as a diagnostic tool to help a dentist decide when to refer a child for an X‐ray examination. The graphs should contribute to earlier detection of unerupted permanent teeth, and recognition of their causes, along with more effective therapy. In children who rank below the 5% percentile curves, the cause for delay should be identified and proper treatment applied. Children who are estimated above 95% or below 5% need special attention from their physician since it is necessary to find the causes of such large deviations.

According to our study, the duration of mixed dentition is at present longer than in children born 30 years ago (Premik et al., 1994) which is in agreement with the study by Eskeli et al. (2016).

In patient care, the prediction of the timing of tooth eruption is useful in interceptive guidance of occlusion. Rowlands et al. (2006) reported that the eruption of mandibular second premolars is of special interest. At first, the exfoliation of the second deciduous molars provides leeway space, which is of major importance in the diagnosis of arch length. Another point to consider is that mandibular second premolars are usually the last successor teeth to erupt in the mandible and can determine the beginning of full orthodontic treatment.

In this study was found that in Class III malocclusion all the permanent mandibular teeth erupt earlier than the maxillary teeth with statistically significant (*p* < .05) differences for all teeth except the second premolars and second molars. Studies by Haruki et al. (1997) and Suda et al. (2002) also showed delayed formation and eruption of the maxillary teeth, especially molars.

The mean eruption times for all the permanent mandibular teeth in Class III malocclusion were lower compared with Class I and Class II malocclusion except for the second premolars and second molars. Study by Brin et al. (2006) suggested that the maxillary second molars might erupt earlier in patients with skeletal Class II malocclusions although tooth eruption has a poor correlation with facial growth. In the present study, we found the mean eruption times for all the permanent maxillary teeth in Class II malocclusion were lower compared with Class I and Class III malocclusion except for the maxillary canine and first molar.

Although this study may not represent the Slovenian population as a whole, the results are useful for pediatric dentists, orthodontists, and health workers.

## 5 CONCLUSION

Teeth have a high functional and esthetic value. It is important to know the normal eruption time of permanent teeth in the population to timely detect dental anomalies such as a congenitally missing tooth or an impacted tooth which can affect scheduling dental and orthodontic treatment. Teeth eruption times are of interest to numerous clinical and public health fields such as pediatric dentistry, orthodontics, anatomy, anthropology, and community medicine for monitoring the growth of children.

However, data from the present study cannot be generalized to all Slovenian children. The longitudinal study design will make it possible to further explore the impact of possible disturbing factors on tooth emergence.

## CONFLICT OF INTERESTS

The author declares that there is no conflict of interests.

## AUTHOR CONTRIBUTIONS

Anita Fekonja—the author has been involved in (1) conception and design of the study, (2) analysis and interpretation of data, (3) writing the article, (4) revising and final approval of the submitted version of the article.

## Data Availability

The data that support the findings of this study are available on request from the corresponding author. The data are not publicly available due to privacy or ethical restrictions.
